# The Relationship between Weight Status, Health-Related Quality of Life, and Life Satisfaction in a Sample of Spanish Adolescents

**DOI:** 10.3390/ijerph17093106

**Published:** 2020-04-29

**Authors:** José Ignacio Baile, Raquel María Guevara, María José González-Calderón, José David Urchaga

**Affiliations:** 1 Faculty of Health Sciences and Education, Madrid Open University, La Coruña Highway, km 38.500, Collado Villalba, 28400 Madrid, Spain; joseignacio.baile@udima.es (J.I.B.); mariajose.gonzalez@udima.es (M.J.G.-C.); 2 Faculty of Education, Pontifical University of Salamanca, Street Henry Collet, 52-70, 37007 Salamanca, Spain; 3 Faculty of Comunication, Pontifical University of Salamanca, Street Henry Collet, 52-70, 37007 Salamanca, Spain; jdurchagali@upsa.es

**Keywords:** body mass index, health-related quality of life, life satisfaction, obesity, overweight

## Abstract

Excess weight has been associated with numerous psychosocial problems and is considered to be one of the most important health problems of today. The aim of this study is to analyze the relationship between weight status, which is evaluated by means of the body mass index (BMI), and the health-related quality of life (HRQoL) and life satisfaction (LS) variables in Spanish adolescents, as well as to examine whether gender influences this interrelationship. A total of 1197 subjects studying in their 1st and 4th years of high school (mean age: 14.4 years, *SD*: 1.69) participated in the study by completing the Health Behavior in School-Aged Children (HBSC) questionnaire. Then, the participants were grouped into the following categories: underweight, normal weight, overweight, and obese. The results show that boys have significantly higher HRQoL as well as higher levels of LS. On the other hand, only the obese group shows significantly lower scores in both HRQoL and LS than those in the normal-weight group. The interaction of weight category and gender does not have a significant impact on the variables that have been analyzed (HRQoL or LS).

## 1. Introduction

In recent decades, there has been a significant increase in the prevalence of overweight and obesity among the Spanish and world population [1], with a particularly significant rise in the child and youth population, as indicated by recent studies [2]. Excess weight has been associated with numerous concerns connected to biomedical [3] and psychosocial orders [4], and it is considered one of the most important health problems facing humanity today [5]. Likewise, the impact of said excess weight, either directly or through other health issues linked to it, can contribute to both a reduced health-related quality of life and lower life satisfaction, as has already been shown in different groups [6,7], including adolescents [8,9,10,11]. More specifically, in adolescence, body image becomes important in the development of one’s self-concept and has an influence on self-esteem and interpersonal relationships with peers [12,13]; for this reason, it is foreseeable that the higher the weight and the worse the body image, the worse the psychosocial indicators will be, and consequently, the subjective or perceived quality of life and life satisfaction.

Regarding life satisfaction (LS), an overall evaluation that people have of their own life [14] or a judgmental process in which individuals assess the quality of their lives on the basis of their own unique set of criteria [15], it seems to be closely related to health and subjective well-being [6]. Particularly, in adolescence, the perception of life satisfaction has important implications for psychological [16,17], social [18], and educational functioning [19,20], and it is associated with family and school satisfaction [21], as well as with other aspects of well-being, most notably health [22]. Specifically, lower life satisfaction has been found among adolescents that are overweight and obese relative to healthy weight youth [23]. As far as gender differences are concerned, several studies have found that teenage boys report significantly higher levels of life satisfaction than teenage girls [22,24,25]. However, a recent meta-analysis of 46 empirical studies revealed that life satisfaction remains invariant across gender groups, with just a slight difference in favor of male adolescents [26]. The negative relationship between obesity and life satisfaction among adolescents has also been found to be stronger for females than males [23].

Health-related quality of life (HRQoL) is a broad concept that can be defined as a patient’s general subjective perception of the effect of illness or medical condition and intervention on physical, psychological, and social aspects of daily life [27]. Therefore, HRQoL includes physical and occupational function, psychological state, social interaction, and somatic sensation [28]. Different cross-national studies have found that female adolescents report significantly lower levels of HRQoL than male adolescents [11,29,30,31]. This gender difference has also been documented in Spanish teenagers [32]. As far as excess weight is concerned, current research has found that compared with normal-weight adolescents, adolescents who are overweight or obese report significantly lower HRQoL regardless of origin [10,11,33,34,35]. Moreover, in this developmental stage, a greater effect of overweight and obesity on their health-related quality of life has been found in females than in males [33,35,36]. However, although the association between excess weight and poor HRQoL has been repeatedly documented in clinical samples of severely obese youth, some researcher such as Farhat et al. suggest that evidence for that association in population-based samples remains inconclusive and that research examining that relationship is scarce [9].

Taking into account the scarcity of data on the above-mentioned variables in the adolescent Spanish population, this study aims to analyze the effect of weight status, as assessed through the body mass index (BMI), on health-related quality of life (HRQoL) and life satisfaction (LS) in a sample of Spanish adolescents. This study also examines whether gender influences these relationships. It is hypothesized that adolescents who are far from a normal weight, in excess or deficit, will present lower rates of both HRQoL and LS.

## 2. Materials and Methods

### 2.1. Design and Participants

A descriptive and cross-sectional study was designed. The research involved 1197 Spanish adolescents (50.3% girls) in the 1st and 4th years of high school (mean age: 14.4 years; *SD*: 1.69). A stratified multistage cluster random sampling method was used to recruit the participants. The margin of sampling error was +/−2.8% (*p* = q = 0.5; confidence level = 95%).

When the participants’ BMI data were coded, they were then grouped into the following four categories, using the Orbegozo [37] foundation tables as a reference: underweight (percentile: <30), normal weight (percentile: 30 < 85), overweight (percentile: ≥85–95), and obese (percentile: >95). The BMI scales according to the participants’ sex and age can be seen in Table 1.

### 2.2. Instruments

The subjects completed the Health Behavior in School-Aged Children (HBSC) questionnaire [38], which evaluated, in addition to sociodemographic variables, the three variables under analysis.

*Weight Category.* This was evaluated using the Body Mass Index (BMI), which was obtained through the weight and height data that was self-reported by the participants and calculated by the following equation: kg/m^2^. The validity of self-reported data in obtaining BMI in studies with children and youth has already been supported in previous research [39,40]. According to their BMI, participants were grouped into the following categories: underweight, normal weight, overweight, and obese.

*Health-Related Quality of Life (HRQoL).* This was evaluated using the Kidscreen-10 [41] instrument included in the HBSC questionnaire, which establishes a global index of health-related quality of life or emotional well-being through responding to 10 items that include physical, psychological, and social aspects of well-being. The child should indicate if “in the last week” he or she has felt good and fit (1), full of energy (2), sad (3), lonely (4), if he or she has had enough time for himself/herself (5), if he or she has been able to do the things he/she wanted in his/her free time (6), if his/her parents have treated him/her fairly (7), if he/she has had fun with his/her friends (8), if he/she has done well in the school (9), and if he/she has been able to concentrate or pay attention (10). Each item is scored on a scale of 1 to 5, which correspond to the following options: never, almost never, sometimes, almost always, and always. Items 1 and 9 vary in the response options as follows: not at all, slightly, moderately, very, and extremely. Items 3 and 4 are corrected in reverse. The total score of the Kidscreen-10, which is obtained by adding the scores of all the items, ranges from between 10 and 50, so the higher the score obtained, the higher the health-related quality of life an individual experiences. This instrument was selected because it is the reference technique used in Europe for assessing HRQoL in the adolescent population, as well as for its adequate psychometric properties [42]; in fact, in this sample, the reliability of the instrument was high (α = 0.795).

*Life Satisfaction (LS).* To evaluate this variable, the Cantril Scale [43] was used, which is also included in the HBSC questionnaire. It consists of the following single item: “In general, in what place do you feel your life is at this moment?” Responses range from 0 (“the worst possible life”) to 10 (“the best possible life”).

### 2.3. Procedure

Once the permissions of the educational centers and the informed consent of the parents or legal guardians were obtained, the HBSC questionnaire was administered within the school’s class schedule by four trained researchers. Previously, both family members and educational leaders were given detailed information on the study and were informed that the data would be kept confidential. Moreover, the subjects, whose participation was voluntary, as well as their families were guaranteed anonymity at all times.

This study meets the ethical principles for medical research involving human subjects contained in the Declaration of Helsinki developed by the World Medical Association in 2013, as well as the Spanish Organic Law 15/1999 of December 13 on the Protection of Personal Data (LOPD) and the Royal Decree 561/1993, of April 16 that regulates the conduct of clinical trials with human subjects.

### 2.4. Data Analysis

In order to determine whether there were differences in the variables under analysis (HRQoL and LS) based on participant gender, t-tests were performed. Additionally, an analysis of variance was carried out after checking the homogeneity between the groups, in order to determine whether there were mean differences in these variables based on the adolescents’ weight category. The homoscedasticity assumption was checked using the Levene’s test. For the post hoc analysis of the differences found, the Tukey’s HSD test was performed. To study the effect of how weight status and gender interact in the variables under analysis, a multivariate ANOVA was carried out. In all the analyses regarding differences, the size of the effect was examined with the corresponding tests. All statistical analyses were performed using SPSS Version 22.0 for Windows.

## 3. Results

### 3.1. Descriptive Analysis

Table 2 shows the sample distribution based on gender and the BMI category to which the participants belong. To determine whether there were differences between boys and girls based on their BMI category, the non-parametric chi-squared test was used. It was concluded that there was no statistically significant relationship between gender and BMI in the current sample (*χ*^2^ χ^2^ = 3.56, gl = 3, *p* = 0.313).

Table 3 shows the results of all the participants, as well as boys and girls separately, for the variables under analysis, i.e., health-related quality of life (HRQoL) and life satisfaction (LS), based on the BMI category to which they belong.

### 3.2. Health-Related Quality of Life and Life Satisfaction Based on Adolescent Gender

Student’s t-test results show that there was a statistically significant difference between boys and girls, with a statistical confidence level of 95%, in the mean health-related quality of life (t (1195) = 6.01, *p* < 0.001), with a moderate effect size (Cohen’s d = 0.35), as well as in life satisfaction (t (1192) = 3.93, *p* < 0.001), with a small effect size (Cohen’s d = 0.23). As shown in Table 3, boys had higher health-related quality of life (39.09 versus 37.04) as well as higher levels of life satisfaction than their female peers (7.99 versus 7.62).

### 3.3. Health-Related Quality of Life and Life Satisfaction Based on Adolescent Weight Status

The ANOVA results show that adolescents’ BMI groups differed both in health-related quality of life [F(3) = 3.33, *p* = 0.019], and life satisfaction [F(3) = 6.70, *p* < 0.001]. Effect sizes for both tests were very low (HRQoL, η^2^ = 0.017; SV, η^2^ = 0.008). Specifically, the Tukey’s HSD test showed the existence of significant differences between only the obesity and normal-weight categories, both in HRQoL (*p* = 0.014 < 0.05) and in LS (*p* = 0.001 < 0.05). Thus, the adolescents who were obese showed mean scores significantly lower in HRQoL than those who belonged to the normal-weight category (35.45 versus 38.18), with a moderate effect size (Cohen’s d = 0.46). Similarly, for life satisfaction, obese adolescents were found to have significantly lower mean scores than those from the normal-weight group (6.89 versus 7.86) having a medium effect size (Cohen’s d = 0.59). Although no significant differences were found between the other BMI groups in either of the two variables studied, the mean obtained by the overweight and underweight groups were lower than those of the normal-weight group, as expected by both HRQoL and LS.

Regarding the effect of the interaction between weight status and adolescent gender on health-related quality of life and life satisfaction, when the effect of the interaction between gender and the BMI category (weight status) of the adolescents on their HRQoL was studied by means of a multivariate ANOVA, no statistically significant relationships were found [F(3) = 0.530, *p* = 0.662]. Likewise, no statistically significant relationship was found when the effect of this interaction on life satisfaction of the participants was analyzed [F(3) = 1.650, *p* = 0.176].

## 4. Discussion

The results obtained in this study of Spanish adolescents partially confirm the first proposed hypothesis since differences in health-related quality of life and life satisfaction are only observed between the obese group (percentile > 95) and the normal-weight group (percentile 30 < 85). The other two groups (underweight and overweight) do not differ significantly from the normal-weight group in the variables studied. Previous research has also found that compared with healthy adolescents, those who are obese report significantly lower HRQoL and LS [10,11,23,32,33,34]. Likewise, the results obtained are consistent with those of other studies in different populations in which the negative impact of body mass index on the psychosocial health of adolescents [44] has been studied such as those that have shown that the higher the BMI, the greater the risk of victimization of the person or a worse self-concept [45], which could explain the lower levels of HRQoL and LS found. In fact, excess weight in the adolescent population has such an impact on lowering their quality of life that in some studies, it has been found that severely obese youth experience a quality of life similar to that of youth with a chronic condition, such as cancer [7,8,46]. In this sense, longitudinal studies are needed to understand the relationship between extreme obesity and HRQoL, as well as the impact of other lifestyle and socioeconomic factors on it [47].

The fact that only obesity, but not being overweight, affects health-related quality of life and life satisfaction in this study can be due to the samples used in most previous studies examining the relationship between excess weight and poor HRQoL and LS. As Farhat et al. pointed out, that association has been documented in clinical samples of obese youth [47], but the evidence for that relationship in population-based samples, as the one in this study, is not clear yet [9]. The prevailing body aesthetic model could also explain why underweight adolescents do not differ from normal-weight adolescents. Although the interpretative logic with respect to health could lead us to consider that a low weight is negative and should affect the integral development of the person, in the Spanish society, having a low weight is a positive value, causing recognition and reinforcement, that can counteract the negative effects on health, thus generating a positive effect on the subjective variables of quality of life and life satisfaction. Thus, only the opposite extreme, obesity, has a negative impact on health-related quality of life and life satisfaction, which may be due to society overvaluing thinness and its generalized “phobia” of obesity. This interpretation is consistent with previous studies that have analyzed what is considered to be attractive and positively valued in Spanish society, whether thinness or, more generally, “non-obesity” [48,49,50,51].

This study on Spanish adolescents also confirms a general tendency for teenage girls to report significantly lower HRQoL [11,29,30,31,32] and lower life satisfaction [22,24,25] than their male peers regardless of origin. However, contrary to what had been hypothesized and what has been repeatedly confirmed not only in adults and the elderly [52] but also in adolescents [33,35,36], Spanish female youths do not report a significantly greater effect of overweight or obesity on their health-related quality of life or their life satisfaction than male youths. The explanation for this phenomenon is unclear, although it is reasonable, since the BMI is not the only factor that influences LS. It could be that these days, external pressure to be thin or fit is experienced in a similar way by both female and male Spanish adolescents. In Spain, the models of beauty or body image may have also been modified for teenage boys to a greater extent than for teenage girls in the last few years, which in turn can generate higher levels of personal dissatisfaction with their bodies, more stress, and more inappropriate behaviors in boys, i.e., regarding eating habits or the consumption of weight loss substances.

The findings of our study indicate that adolescents with obesity have a worse perceived quality of life and lower life satisfaction than their peers, which indicates the importance of developing the following policies: (1) increasing efforts in the prevention and treatment of childhood and adolescent obesity promoting physical exercise, since recent research has pointed out the pathological consequences of lack of adequate exercise [53]; (2) ensuring safe, healthy, and nurturing school environments and promoting school-based interventions which have been probed to be effective in changing not only knowledge and attitudes but also behaviors, healthy policies, and environments [54]; (3) implementing educational and social measures to avoid discrimination against people with obesity, especially emphasizing the child and adolescent stages, in which the effects of discrimination are more dangerous; and (4) promoting healthy aesthetic models that correspond to the real shapes and weight of the population, avoiding idealizing thinness.

Regarding the limitations of the study, this could have been more complete if objective evaluations of the health-related quality of life of the participants or the assessment of their body image had been incorporated, or if an exploration of how these individuals develop socially had been carried out. These aspects will be taken into account in future research.

## 5. Conclusions

In conclusion, the results of this research paper indicate the following: (a) compared with healthy adolescents, obese report significantly lower HRQL and LS; (b) the body mass index has a negative impact on the psychosocial health of adolescents: the higher the BMI, the greater the risk of victimization of the person or a worse self-concept; (c) excess weight in the adolescent population has such an impact on lowering their quality of life; (d) teenage girls report significantly lower HRQoL and lower life satisfaction.

This study shows the importance of increasing efforts in the prevention of childhood and adolescent obesity, implementing educational and social measures to avoid discrimination against people with obesity and promoting healthy aesthetic models that correspond to the real shapes and weight of the population.

## Figures and Tables

**Table 1:** Body mass index according to the subjects’ sex and age.

	Boys			Girls		
Age	P30	P85	P95	P30	P85	P95
12	17.02	22.36	24.41	17.35	22.84	24.99
12.5	17.27	22.71	24.82	17.62	23.08	25.21
13	17.51	23.08	25.23	17.97	23.32	25.42
13.5	17.87	23.47	25.67	17.23	23.58	25.67
14	18.11	23.90	26.16	18.48	2.88	25.95
14.5	18.45	24.37	26.68	18.88	24.21	26.26
15	18.78	24.85	27.21	19.17	24.56	26.61
15.5	19.06	25.33	27.74	19.50	24.91	26.96
16	19.37	25.78	28.22	19.81	25.19	27.24
16.5	19.50	26.11	28.57	19.88	25.38	27.43
17	19.73	26.25	28.70	19.96	25.36	27.39
17.5	19.68	26.07	28.47	19.73	25.00	26.98
18	19.65	25.43	27.71	19.52	24.16	26.04

**Table 2:** Distribution of the sample according to the weight status of the participants.

Sex	*N* (%)	Weight Status				*χ* ^2^
		Underweight	Normal Weight	Overweight	Obese	*P* ^C^
Boys	590 (49.3%) ^A^	14 (2.4%) ^B^	520 (88.2%) ^B^	28 (4.7%) ^B^	28 (4.7%) ^B^	0.313
Girls	607 (50.7%) ^A^	20 (3.3%) ^B^	543 (89.4%) ^B^	26 (4.3%) ^B^	18 (3.0%) ^B^	
Total	1197 (100%) ^A^	34 (2.8%) ^B^	1063 (88.9%) ^B^	54 (4.5%) ^B^	46 (3.8%) ^B^	

^A^: Percentage by Colum; ^B^: Percentage by Row; ^C^: Comparasion between boys and girls.

**Table 3:** Health-related quality of life (HRQoL) and life satisfaction (LS) based on gender and weight status of the participants.

Variable	Weight Status	Boys		Girls		Total		Comparisons
		Mean	SD	Mean	SD	Mean	SD	
HRQoL	Underweight	38.21	6.69	36.65	5.18	37.29	5.81	Girls < Boys ***
	Normal weight	39.33	5.61	37.09	6.07	38.18	5.95	Obesity < Normal weight *
	Overweight	38.50	5.77	37.77	7.73	38.15	6.73	
	Obesity	35.71	6.87	35.06	5.32	35.45	6.26	
	Total	39.09	5.75	37.04	6.10	38.05	6.01	
LS	Underweight	8.00	1.66	7.50	2.12	7.71	1.93	Girls < Boys ***
	Normal weight	8.06	1.52	7.66	1.66	7.86	1.60	Obesity < Normal weight *
	Overweight	7.36	1.75	7.77	1.45	7.56	1.61	
	Obesity	7.29	2.02	6.28	2.02	6.89	2.06	
	Total	7.99	1.57	7.62	1.69	7.80	1.64	

* *p* < 0.05; *** *p* < 0.001.
